# ImmunoPET Imaging Identifies the Optimal Timepoint for Combination Therapy in Xenograft Models of Triple-Negative Breast Cancer

**DOI:** 10.3390/cancers15051589

**Published:** 2023-03-03

**Authors:** Ziqi Li, Erika Belitzky, Ondrej Blaha, Alessandra Cavaliere, Samantha R. Katz, Mariam Aboian, Lindy Melegari, Khashayar Rajabimoghadam, Stephen Kurpiewski, Xiaohua Zhu, Bernadette Marquez-Nostra

**Affiliations:** 1 Department of Nuclear Medicine, Tongji Hospital, Tongji Medical College, Huazhong University of Science and Technology, Wuhan 430030, China; 2 Department of Radiology and Biomedical Imaging, Yale University, New Haven, CT 06520, USA; 3 Yale Center for Analytical Sciences, Yale School of Public Health, Yale University, New Haven, CT 06520, USA; 4 Department of Radiology, University of Alabama at Birmingham, Birmingham, AL 35233, USA

**Keywords:** gpNMB, CDX-011, triple-negative breast cancer, dasatinib, PET imaging, ^89^Zr, upregulation

## Abstract

**Simple Summary:**

Different combination therapies are being investigated for the treatment of triple-negative breast cancer (TNBC). Dasatinib is one such drug that targets the Src pathway to kill cancer cells. However, dasatinib treatment can also increase the expression of a different biomarker called glycoprotein non-metastatic melanoma B (gpNMB), in which high levels of gpNMB on the surface of TNBC cells is associated with lower overall survival of patients. This effect can be exploited to enhance the delivery of other drugs carried by antibodies that bind to gpNMB, known as antibody drug conjugates. Determining when dasatinib increases gpNMB expression is important for treatment planning. The aims of our study are two-fold. The first is to evaluate how much and when does dasatinib increase gpNMB expression through noninvasive imaging of gpNMB in mice models of TNBC. The second aim is to explore the efficacy of the combination treatment with gpNMB-targeted antibody drug conjugate and dasatinib in shrinking the tumor. These findings can be used for image-guided approaches for combination treatments in TNBC.

**Abstract:**

(1) Purpose: The glycoprotein non-metastatic melanoma B (gpNMB) is a type 1 transmembrane protein that is overexpressed in numerous cancers, including triple-negative breast cancer (TNBC). Its overexpression is associated with lower overall survival of patients with TNBC. Tyrosine kinase inhibitors such as dasatinib can upregulate gpNMB expression, which has the potential to enhance therapeutic targeting with anti-gpNMB antibody drug conjugates such as glembatumumab vedotin (CDX-011). Our primary aim is to quantify the degree and identify the timeframe of gpNMB upregulation in xenograft models of TNBC after treatment with the Src tyrosine kinase inhibitor, dasatinib, by longitudinal positron emission tomography (PET) imaging with the ^89^Zr-labeled anti-gpNMB antibody ([^89^Zr]Zr-DFO-CR011). The goal is to identify the timepoint at which to administer CDX-011 after treatment with dasatinib to enhance therapeutic efficacy using noninvasive imaging. (2) Methods: First, TNBC cell lines that either express gpNMB (MDA-MB-468) or do not express gpNMB (MDA-MB-231) were treated with 2 μM of dasatinib in vitro for 48 h, followed by Western blot analysis of cell lysates to determine differences in gpNMB expression. MDA-MB-468 xenografted mice were also treated with 10 mg/kg of dasatinib every other day for 21 days. Subgroups of mice were euthanized at 0-, 7-, 14-, and 21-days post treatment, and tumors were harvested for Western blot analysis of tumor cell lysates for gpNMB expression. In a different cohort of MDA-MB-468 xenograft models, longitudinal PET imaging with [^89^Zr]Zr-DFO-CR011 was performed before treatment at 0 (baseline) and at 14 and 28 days after treatment with (1) dasatinib alone (2) CDX-011 (10 mg/kg) alone, or (3) sequential treatment of dasatinib for 14 days then CDX-011 to determine changes in gpNMB expression in vivo relative to baseline. As a gpNMB-negative control, MDA-MB-231 xenograft models were imaged 21 days after treatment with dasatinib, combination of CDX-011 and dasatinib, and vehicle control. (3) Results: Western blot analysis of MDA-MB-468 cell and tumor lysates showed that dasatinib increased expression of gpNMB in vitro and in vivo at 14 days post treatment initiation. In PET imaging studies of different cohorts of MDA-MB-468 xenografted mice, [^89^Zr]Zr-DFO-CR011 uptake in tumors (SUV_mean_ = 3.2 ± 0.3) was greatest at 14 days after treatment initiation with dasatinib (SUV_mean_ = 4.9 ± 0.6) or combination of dasatinib and CDX-011 (SUV_mean_= 4.6 ± 0.2) compared with that at baseline (SUV_mean_ = 3.2 ± 0.3). The highest tumor regression after treatment was observed in the combination-treated group with a percent change in tumor volume relative to baseline (%CTV) of −54 ± 13 compared with the vehicle control-treated group (%CTV = +102 ± 27), CDX-011 group (%CTV = −25 ± 9.8), and dasatinib group (%CTV = −23 ± 11). In contrast, the PET imaging of MDA-MB-231 xenografted mice indicated no significant difference in the tumor uptake of [^89^Zr]Zr-DFO-CR011 between treated (dasatinib alone or in combination with CDX-011) and vehicle-control groups. (4) Conclusions: Dasatinib upregulated gpNMB expression in gpNMB-positive MDA-MB-468 xenografted tumors at 14 days post treatment initiation, which can be quantified by PET imaging with [^89^Zr]Zr-DFO-CR011. Furthermore, combination therapy with dasatinib and CDX-011 appears to be a promising therapeutic strategy for TNBC and warrants further investigation.

## 1. Introduction

Breast cancer is the most common cancer diagnosed in women [1]. In 2022, breast cancer accounted for 31% of cancer diagnoses and was the leading cause of cancer-related deaths among women 20–59 years old [1]. A subtype of breast cancer is triple-negative breast cancer (TNBC), which accounts for 10–30% of all breast cancer diagnoses. TNBC is defined by the lack of protein expression of estrogen receptor (ER), progesterone receptor (PR), and human epidermal growth factor receptor 2 (HER2), which are commonly targeted for therapy [2]. Recently, antibodies have been approved for immunotherapy targeting the programmed death receptor ligand 1 (PDL1) or to deliver a cytotoxic drug as an antibody drug conjugate (ADC) that targets the trophoblast receptor 2 (TROP2) [3,4]. Despite these new targeted treatments, TNBC is still associated with an increased risk for metastases and shorter post-recurrence survival [5]. Therefore, there is a need to explore alternative therapeutic targets and identify prognostic markers to select patients likely to benefit from targeted therapy.

An exploratory target for the treatment of TNBC is glycoprotein non-metastatic melanoma B (gpNMB) [6]. gpNMB is a type 1 transmembrane protein that is overexpressed in numerous cancers, including 30–41% of TNBC [7]. Normally located intracellularly, gpNMB is overexpressed on the cell surface of cancer cells, making it an ideal target for ADCs [7]. Glembatumumab vedotin (CDX-011) is an ADC that binds to gpNMB. CDX-011 is a fully human antibody that is linked to a protease-cleavable microtubulin inhibitor, monomethyl auristatin E (MMAE) [8,9,10,11]. CDX-011 showed promising efficacy in preclinical studies and in early phase clinical trials for the treatment of metastatic TNBC [10,12]. However, Phase IIB trial failed clinical endpoints due to resistance to the MMAE payload in some patients [12]. One approach to avoid resistance to ADC therapy is a combination therapy that acts on different signaling pathways of the cancer cell [13,14]. Another approach is to increase gpNMB expression to efficiently deliver the MMAE payload and sensitize the tumor cells to ADC treatment [15].

One potential combination strategy is the use of dasatinib, a tyrosine kinase inhibitor, to increase gpNMB expression and enhance the targeting of gpNMB with CDX-011 [16]. Dasatinib is a tyrosine kinase inhibitor of the Src family. Dasatinib is expected to increase the expression of gpNMB and downregulate the expression of phosphorylated Src (p-Src) in a cell while having no effect on the expression of Src [17]. Dasatinib has been approved for the treatment of chronic myeloid leukemia (CML) and is under investigation for other cancers, including breast cancer [18,19,20]. However, there is a need for prognostic markers to help select patients likely to benefit from combination therapy and to identify the timepoint for administering combination treatment based on their mechanisms of action.

Positron emission tomography (PET) imaging is a noninvasive imaging modality that has the potential to predict which patients are likely to respond to treatment by determining the relative expression of therapeutic targets prior to treatment. ImmunoPET combines the specificity of antibodies with the sensitivity of PET imaging to detect antigen biomarkers. ImmunoPET can provide information on the heterogeneity of antigen expression in tumor lesions throughout the whole body [21]. Greater uptake of the radiolabeled antibody corresponds to greater expression of the antigen [22,23]. Previously, we developed [^89^Zr]Zr-DFO-CR011 to image gpNMB in vivo prior to treatment with CDX-011, in which we showed that it could predict early response to CDX-011 monotherapy in preclinical models of TNBC [23,24]. As an extension to this work, our primary aim in the current study is to evaluate the ability of [^89^Zr]Zr-DFO-CR011 to quantify the upregulation of gpNMB expression over the course of treatment with dasatinib or a combination of dasatinib and CDX-011 in gpNMB-positive MDA-MB-468 xenograft models. To achieve this aim, we designed sequential, longitudinal PET studies to monitor changes in gpNMB expression in gpNMB-positive MDA-MB-468 and in gpNMB-negative MDA-MB-231 xenograft models by PET imaging with [^89^Zr]Zr-DFO-CR011. Longitudinal imaging of the antigen throughout the whole body has the potential to monitor how the expression of a therapeutic target changes with a single agent or combination treatment. Secondly, we explored the therapeutic efficacy of each investigational treatment (dasatinib, CDX-011, and their combination) in these animal models of TNBC.

## 2. Materials and Methods

### 2.1. Cell Lines

All cell lines were purchased from the American Type Culture Collection (ATCC, Manassas, VA, USA) and cultured as recommended by ATCC. Cell lines were authenticated by ATCC before and after studies using short tandem repeat (STR) profiling.

### 2.2. Evaluation of gpNMB Expression in the Presence of Dasatinib In Vitro

A 10 mM stock solution of dasatinib (BMS-354825, Selleckchem, Houston, TX, USA) was dissolved in 50% propylene glycol in water prior to diluting in a complete cell medium. MDA-MB-468 and MDA-MB-231 cell lines were treated with 2 µM of dasatinib. This concentration of dasatinib was determined by a dose-finding study in vitro, where MDA-MB-468 and MDA-MB-231 cells were treated with various concentrations of dasatinib for 48 h. Following the treatment, viability assays were performed, and IC_50_ values were determined to be 2.3 μM for MDA-MB-468 and 0.19 μM for MDA-MB-231 cells (Figure S1). Methods for characterizing cell viability and dose finding are available in Supplementary Information. Cells were harvested and lysed for Western blot analysis of gpNMB expression as described in Section 2.3.

### 2.3. Lysate Preparation and Western Blotting of gpNMB Expression

Cells or tumors were homogenized and lysed in RIPA buffer (Sigma-Aldrich, St. Louis, MO, USA, cat# R0278) containing protease inhibitor cocktail with EDTA (ThermoFisher Scientific, Waltham, MA, USA, cat# 78430). The solution was centrifuged, and the supernatant (lysates) was collected. Total protein was quantified in lysates using a BCA protein quantification kit (ThermoFisher cat# 23225).

Protein lysates were separated into 4–20% TGX Stain-Free Gels (Mini-Protean). Protein bands were electroblotted onto PVDF membranes at 50 V for 2 h. Membranes were sliced into two pieces to stain the top part of the membrane for gpNMB (75–125 kDa), Src (60 kDa), or p-Src (60 kDa), while the bottom part was immunoblotted for β-actin (42 kDa). Nonspecific binding was blocked with 3% bovine serum albumin (BSA) in TBST, and proteins of interest were probed with the following primary antibodies at 4 °C overnight: goat-anti-human gpNMB (0.5 µg/mL, AF2550 R&D systems); mouse anti-Beta-actin (1:5000, MA1-140, ThermoFisher); rabbit anti-Src (1:500, Cell Signaling Technology, Danvers, MA, USA, cat# 2109); and rabbit anti-Phospho-Src Family (Tyr416) (1:1000, Cell Signaling cat# 6943). The membranes were washed with TBST 3x and subsequently probed with the corresponding HRP-conjugated secondary antibodies: Donkey anti-goat IgG (1:15,000, Abcam, Boston, MA, USA, cat# ab97110); anti-mouse IgG (1:10,000, Cell Signaling cat# 7076S); and anti-rabbit IgG (1:3000, Cell Signaling cat# 7074). Protein bands were developed using a west pico substrate kit following supplier instructions (ThermoFisher cat# 34579). Blots were imaged using BOX:9 (Syngene International Ltd, Bangalore, India). The intensity of protein bands was quantified using Image J (imagej.net) and those for gpNMB, p-Src, and Src were normalized to beta-actin.

### 2.4. Xenograft Models

All animal studies were performed in accordance with a protocol approved by the Yale Animal Care and Use Committee. Female athymic nude mice (5–8 weeks old) were obtained from Charles River Laboratories. Xenografts were generated by inoculating the mammary fat pad of mice with 100 µL of 5 × 10^6^ MDA-MB-468 or 1 × 10^6^ MDA-MB-231 cells suspended in 30% Matrigel© Basement Membrane Matrix (Corning, Corning, NY, USA) in PBS.

### 2.5. Determining the Timepoint of gpNMB Upregulation after Dasatinib Treatment in MDA-MB-468 Xenograft Models by Western Blot Analysis of Tumor Lysates

MBA-MB-468 xenografts were administered oral dasatinib formulated in 50% propylene glycol in water at a dose of 10 mg/kg or vehicle control every other day for 14 days. This dose was selected based on the 2.3 μM IC_50_ value of dasatinib for MDA-MB-468 cells in vitro (Figure S1) and a previous study [25]. Subgroups of mice (n = 3 per group) were euthanized at 0 (baseline), 7-, 14-, and 21-days post-treatment initiation. Tumors were harvested, lysed, and Western blots for gpNMB expression were performed as described above (Section 2.3).

### 2.6. Conjugation and Radiolabeling of CR011

**Scheme 1:**
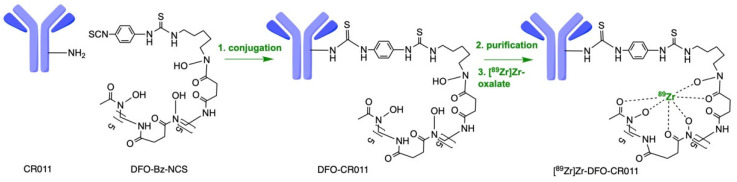
Conjugation and radiolabeling of CR011. (1) Conjugation conditions: The mixture of 5 mol excess of DFO-Bz-NCS to CR011 antibody in PBS pH 9 was reacted at 37 °C for 1 h. (2) Purification: Zeba desalting column (40 MWCO) in 10 mM sodium citrate pH 6.8. (3) Radiolabeling: Neutralized [^89^Zr]Zr-oxalate was added to DFO-CR011 at a specific activity of 167 MBq/mg.

The anti-gpNMB CR011 antibody (Celldex Therapeutics, Phillipsburg, NJ, USA) was conjugated and radiolabeled with neutralized [^89^Zr]Zr-oxalate to obtain [^89^Zr]Zr-DFO-CR011 following our previous studies [24]. A five-fold molar excess of desferrioxamine-p-benzyl-isothiocyanate metal chelate (DFO-Bz-NCS, MW = 752.9 g/mol, Macrocyclics, Inc., Plano, TX, USA) was conjugated to CR011 antibody (MW = 150 kDa, provided in kind by Celldex Therapeutics) in phosphate-buffered saline (PBS, pH = 9) at 37 °C for 1 h. The DFO-CR011 conjugate was buffer exchanged in 10 mM sodium citrate buffer (pH 6.8) using Zeba spin desalting columns (MWCO = 40 kDa). The concentration of DFO-CR011 was determined using a BCA kit (ThermoFisher). DFO-CR011 was then reacted with neutralized [^89^Zr]Zr-oxalate (Washington University in St. Louis, St. Louis, MO, USA, or 3D Imaging, Little Rock, AR, USA) at 37 °C for 1 h to obtain [^89^Zr]Zr-DFO-CR011 for a target-specific activity of 167 MBq/mg. Radiochemical yield was determined via radio-TLC using 50 mM DTPA (pH = 7) as the mobile phase. Once the radioconjugate reaches at least 95% radiochemical yield as determined by radio-TLC, then the radioconjugate is buffer exchanged in 2 mM sodium citrate in saline and diluted further in sterile saline to obtain a final concentration of 18.5 MBq/mL of the tracer for animal studies. Scheme 1 shows the conjugation and radiolabeling of CR011. Protein aggregation analysis was also performed on the final formulation via radio-SEC-HPLC (TSKgel SuperSW3000, TOSOH Corporation, Tokyo, Japan) using the mobile phase consisting of 50 mM sodium phosphate, 150 mM sodium chloride, and 0.1% Tween-20 (pH 7) at a flow rate of 0.25 mL/min.

### 2.7. PET Imaging Acquisition

For small animal PET imaging, mice were anesthetized using isoflurane (1.5–2% *v/v* in O_2_). A radioactivity dose of 1.4 ± 0.28 MBq of [^89^Zr]Zr-DFO-CR011 in 100 μL solution (specific activity = 150 MBq/mg, protein dose = 9.2 ± 1.9 g) was injected via the tail vein, and PET imaging was performed at 7 days post injection (p.i.). Static PET images were acquired for 20 min, followed by 10 min of CT image acquisition using an Inveon PET/CT scanner (Siemens, Knoxville, TN, USA). Images were reconstructed using an ordered subset expectation maximization (OSEM-3D) algorithm and co-registered with CT images using the Inveon Research Workplace Workstation software (Siemens). Regions of interest (ROI) were drawn on the tumor and normal organs, including heart, lungs, liver, and bone regions, using the Inveon Research Workplace Workstation software. The uptake of the radiotracer was quantified by calculating the mean standardized uptake values (SUV_mean_) for each organ and tumor-to-heart SUV ratios from decay-corrected ROI activity concentrations.

### 2.8. PET Imaging and Treatment Paradigm of gpNMB-Positive MDA-MB-468 Xenograft Models

**Figure 1:**
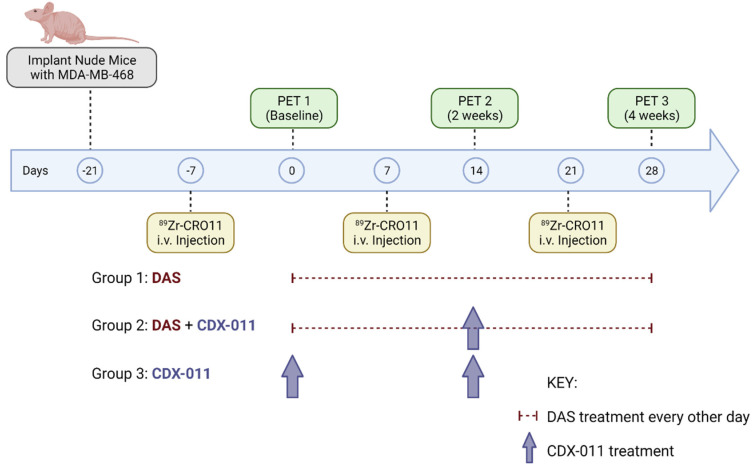
PET imaging and treatment paradigm in MDA-MB-468 xenografted mice. PET imaging with [89Zr]Zr-DFO-CR011 was performed at baseline, 14 days, and 28 days after treatment with dasatinib, CDX-011, or a combination of dasatinib and CDX-011 to monitor changes in gpNMB expression in vivo. DAS = dasatinib. A subgroup of mice (n = 4 out of 8 total) in each treatment arm underwent both PET imaging and therapy, while remaining mice only underwent therapy.

The imaging and treatment paradigm for the gpNMB-positive MDA-MB-468 xenografted mice are outlined in Figure 1. PET imaging began at 4 weeks post inoculation of MDA-MB-468 cells when tumor volumes ranged from 100 to 500 mm^3^. PET imaging with [^89^Zr]Zr-DFO-CR011 was performed 7 days p.i. of the tracer (baseline). Mice were subsequently randomized for treatment with dasatinib (n = 16), PBS (n = 6), or CDX-011 (n = 8) for 14 days. Then, the mice treated with dasatinib were separated into two groups (n = 8 per group), where one group continued with dasatinib treatment alone while the other received the combination of dasatinib and CDX-011 for another 14 days. Dasatinib treatment was administered via oral gavage at a dose of 10 mg/kg every other day for a total of 28 days. CDX-011 treatment was administered via intraperitoneal injection at a dose of 10 mg/kg after baseline PET imaging and at 14 days post treatment initiation. PET imaging with [^89^Zr]Zr-DFO-CR011 was repeated 14 and 28 days after treatment initiation to determine changes in gpNMB expression in vivo over the course of therapy. For this longitudinal study, [^89^Zr]Zr-DFO-CR011 was injected a total of three times in the same animals (Figure 1). Tumor volumes were measured twice per week up to 28 days after treatment initiation and calculated as length × width × height × 0.5. The percent change in tumor volume (%CTV) for each mouse was calculated relative to its initial tumor volume at baseline, where negative values indicate tumor shrinkage and positive values indicate tumor growth after treatment. The health of mice during the treatment period was monitored twice per week using body-condition scoring (BCS) [26].

### 2.9. PET Imaging and Treatment Paradigm of gpNMB-Negative MDA-MB-231 Xenograft Models

**Figure 2:**
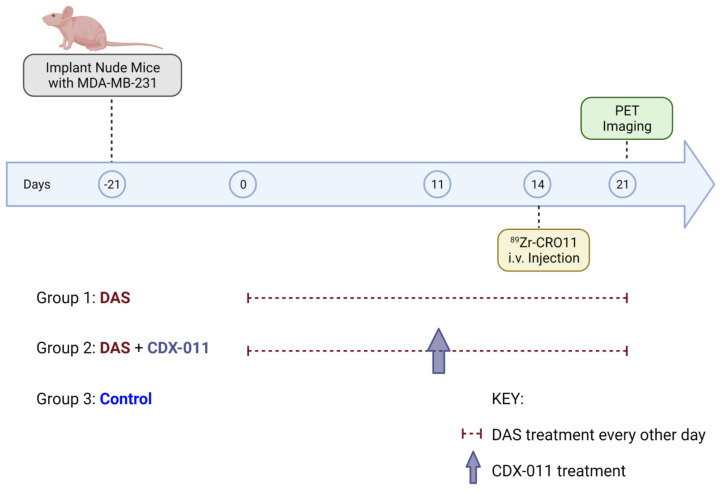
PET imaging and treatment paradigm in gpNMB-negative MDA-MB-231 xenografted mice. PET imaging with [^89^Zr]ZrDFO-CR011 was performed at 21 days after treatment of dasatinib (n = 4), the combination of dasatinib and CDX-011 (n = 5), or vehicle control group (n = 4). DAS = dasatinib. Figure created with Biorender.com.

As gpNMB-negative controls, mice with MDA-MB-231 xenografts were randomized and treated with (1) dasatinib, (2) a combination of dasatinib and CDX-011, or (3) PBS control. Dasatinib treatment was administered at a dose of 10 mg/kg every other day for 21 days. This dose was selected based on a previous study [27]. For the combination group, CDX-011 was given at 10 mg/kg at 11 days post treatment initiation. PET imaging on MDA-MB-231 xenografted mice was performed 7 days p.i. of [^89^Zr]Zr-DFO-CR011 at 21 days after treatment initiation. For this gpNMB-negative control group, the tracer was only administered once, with imaging performed at the conclusion of each treatment regimen (Figure 2). Tumor volumes were measured twice per week up to 21 days after treatment initiation, as described in Section 2.5. The %CTV was also calculated as described in Section 2.8. Treatment began at 2 weeks post inoculation of MDA-MB-231 cells when tumor volumes ranged from 100 to 500 mm^3^ at treatment initiation (Figure 2).

### 2.10. Statistical Evaluation

Data were analyzed using R version 4.1.2 (The R Foundation for Statistical Computing). The outcome variable (%CTV) is expressed as mean (S.D.). The efficacy of the treatment was analyzed using a Kruskal–Wallis test at the final time point/upon trial completion. Pairwise Wilcoxon Rank Sum Test with Benjamini–Hochberg correction for multiple comparison was used posthoc to determine the differences between the individual pairs of treatment. The level of significance was set at α = 0.05. For other comparisons of means, a standard *t*-test was used for the evaluation of two groups, a one-way ANOVA for three or more groups testing one variable, and a two-way ANOVA for two groups with two variables. For ANOVA, Bonferroni posthoc analyses were performed. A *p*-value of < 0.05 was considered significant. GraphPad Prism version 9.4.1 (Boston, MA, USA) was used for graphical analyses.

## 3. Results

### 3.1. Dasatinib Treatment Upregulates gpNMB Expression in MDA-MB-468 Cells In Vitro

**Figure 3:**
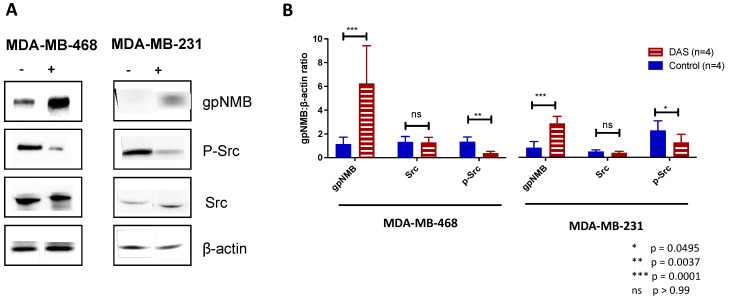
Dasatinib can upregulate the expression of gpNMB on MDA-MB-468 and MDA-MB-231 cells in vitro. (**A**) Western blot for gpNMB, ꞵ-actin, and dasatinib-related proteins p-Src, Src; (**B**) Normalized gpNMB expression relative to ꞵ-actin. DAS = dasatinib.

The cell line MDA-MA-468 was chosen due to its moderate expression of gpNMB; MDA-MB-231 was selected because it does not express gpNMB and was used as a negative control [23]. Both cell lines were treated with 2.3 μM of dasatinib for 48 h. Cells were lysed, and Western blot analyses were performed thereafter to evaluate the effects of dasatinib on the expression of gpNMB, Src, and p-Src (Figure 3). All Western blot images can be found in the supplemental information (Figure S2). The expression of gpNMB increased by 440% (*p* = 0.0001) in the MDA-MB-468 cell line. Interestingly, gpNMB expression was induced in MDA-MB-231 cells but was overall 75% lower than in dasatinib-treated MDA-MB-468 cells. A significant decrease in p-Src expression was observed in both MDA-MB-468 (72% decrease, *p* = 0.0037) and MDA-MB-231 (45% decrease, *p* = 0.0495), which confirmed the mechanism of action for dasatinib therapy. As expected, there was no change in Src expression for either MDA-MB-468 (*p* > 0.9999) or MDA-MB-231 (*p* = 0.992). Statistical significance was based on a two-way ANOVA analysis.

### 3.2. gpNMB Upregulation Was Determined to Be 14 Days after Dasatinib Treatment In Vivo

**Figure 4:**
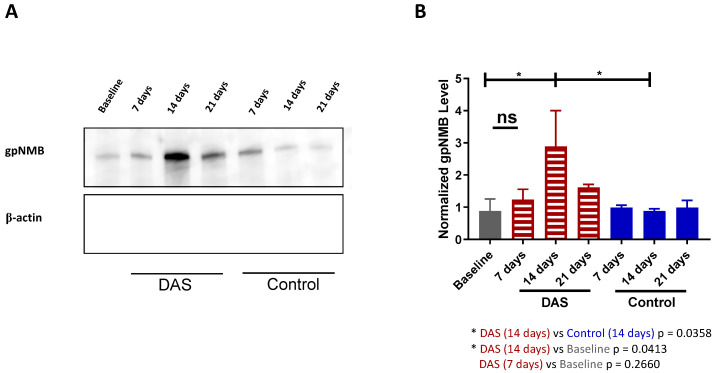
Dasatinib can upregulate the expression of gpNMB in MDA-MB-468-bearing mice. The mice were separated into two groups and were treated with either dasatinib or vehicle control for 21 days. Subgroups of mice (n = 4) were euthanized at different timepoints. Tumors were then subjected to Western blot analyses of gpNMB and ꞵ-actin. (**A**) Western blot images of gpNMB and ꞵ-actin; (**B**) Plot of normalized gpNMB expression with ꞵ-actin. DAS = dasatinib. ns = not significant.

gpNMB upregulation was further investigated in vivo by the treatment of MDA-MB-468 xenografts with dasatinib or vehicle control. Subgroups of mice (n = 3 per group) were euthanized at different timepoints, and tumors were harvested and lysed at 0 (baseline), 7, 14, and 21 days after treatment for Western blot analysis of gpNMB expression (Figure 4). All Western blot images can be found in Supplementary Material (Figure S2). Band intensity analysis showed a 3-fold increase in gpNMB expression at 14 days post-treatment initiation compared with that at baseline (*p* = 0.0413) and with that of the vehicle control group (*p* = 0.0358). The expression of gpNMB at 7- and 21-days post-treatment initiation was not significantly different from that at baseline (*p* = 0.2660, two-way ANOVA analysis).

### 3.3. PET Imaging with [^89^Zr]Zr-DFO-CR011 Demonstrated Increased Tracer Uptake in MDA-MB-468 Tumors In Vivo at 14 Days after Dasatinib Treatment

First, pilot scans with sequential injections of [^89^Zr]Zr-DFO-CR011 in MDA-MB-468 xenografted mice (n = 2) seven days apart were performed to determine the feasibility of serial PET imaging with this tracer. There was no significant difference in the tumor uptake of this tracer between the two injections based on a *t*-test, suggesting that tumor uptake of the tracer from the first injection does not block tumor uptake in the second injection (*p* > 0.05, Figure S3). Then, sequential PET imaging with [^89^Zr]Zr-DFO-CR011 was performed in a different cohort of MDA-MB-468 xenografted mice to determine changes in the level of gpNMB expression over the course of treatment with (1) dasatinib, (2) CDX-011, (3) dasatinib plus CDX-011 combination, or (4) vehicle control. A total of eight mice per treatment group was used. Figure 1 illustrates the PET imaging and treatment paradigm in MDA-MB-468 xenografted mice. Longitudinal PET imaging was performed for the dasatinib, CDX-011, and combination of dasatinib and CDX-011 treatment groups (n = 4 per group) at baseline and at 14 and 28 days after treatment using freshly synthesized [^89^Zr]Zr-DFO-CR011 for each scan.

**Figure 5:**
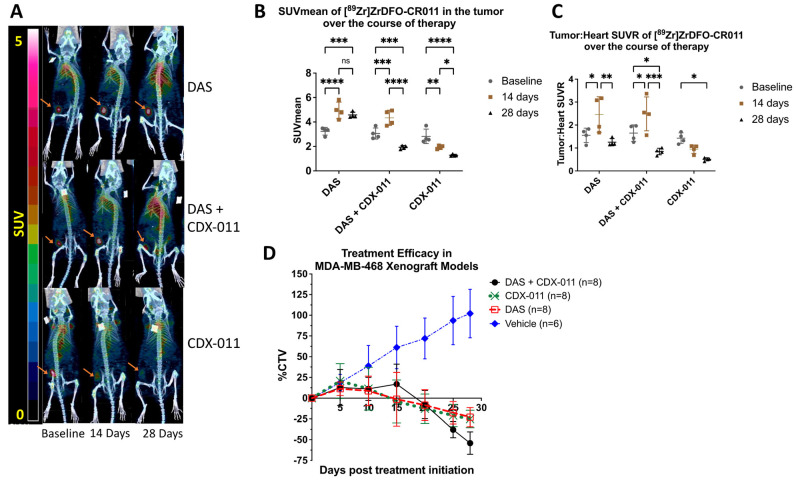
PET/CT imaging with [^89^Zr]Zr-DFO-CR011 can monitor gpNMB expression in MDA-MB-468 xenografted mice noninvasively over the course of treatment with dasatinib (DAS), CDX-011, or their combination. (**A**) Representative PET/CT images as maximum intensity projections from each treatment group over time (n = 4 per group). Arrows point to tumors. RapID ear tags are shown as white squares. (**B**) Quantification of [^89^Zr]Zr-DFO-CR011 uptake in the tumor as SUV_mean_ and (**C**) Tumor-to-Heart SUVR at baseline, 14, and 28 days post treatment initiation. * *p* < 0.05, ** *p* < 0.05, *** *p* = 0.005 **** *p* < 0.0005, ns is *p* > 0.05 (**D**) Change in tumor size measured as the percentage change in tumor volume (%CTV) relative to baseline after treatment initiation (n = 6–8 per group). Vehicle control vs. DAS *p* < 0.0001; Vehicle control vs. CDX-011 *p* < 0.0001; Vehicle control vs. DAS + CDX-011 *p* < 0.0001; DAS vs. CDX-011 *p* = 0.9996; DAS + CDX-011 vs. CDX-011 *p* < 0.05; DAS + CDX-011 vs. DAS *p* < 0.05.

Representative images of the same mice from each treatment group over time are shown as maximum intensity projections, which show elevated uptake of [^89^Zr]Zr-DFO-CR011 in the dasatinib and dasatinib plus CDX-011 combination groups at 14 days post treatment initiation relative to baseline (Figure 5A). Quantification of tracer uptake as SUV_mean_ in the tumor (Figure 5B) and as tumor-to-heart ratios (Figure 5C) was determined for each treatment group at each imaging timepoint. Figure S4 shows the uptake of the tracer in normal organs (heart, lung, liver, bone) over time for each treatment group. These organs were chosen based on the analyst’s confidence in CT-guided image segmentation. PET imaging over time in the heart, lung, and liver showed significantly increased tracer uptake on the third PET imaging time point (28 days post treatment initiation) compared with earlier PET imaging time points (*p* < 0.005, Figure S4).

For the dasatinib-treated group, the SUV_mean_ of [^89^Zr]Zr-DFO-CR011 in the tumor was 3.2 ± 0.3 at baseline, which increased significantly to 4.9 ± 0.7 at 14 days (*p* < 0.001) and was maintained at 4.6 ± 0.23 at 28 days post-treatment initiation (Figure 5B). There was no statistically significant difference between SUV_mean_ at 14 days and that at 28 days post-treatment initiation (*p* > 0.05). Tumor-to-heart ratios were 1.6 ± 0.31 at baseline, which increased to 2.5 ± 0.77 at 14 days (*p* = 0.0173) but then decreased to 1.3 ± 0.16 at 28 days (*p* = 0.0026, two-way ANOVA) post-treatment initiation (Figure 5C). This study demonstrated that immunoPET imaging could monitor changes in gpNMB expression in gpNMB-positive MDA-MB-468 xenograft models and that gpNMB was upregulated at 14 days post treatment initiation with dasatinib.

For the CDX-011-treated group, the SUV_mean_ of [^89^Zr]Zr-DFO-CR011 in the tumor was 2.8 ± 0.6 at baseline, which decreased significantly to 1.9 ± 0.1 at 14 days (*p* = 0.0043) then further decreased to 1.3 ± 0.1 at 28 days (*p* = 0.0291) post-treatment initiation (Figure 5B). The tumor-to-heart ratios followed the same trend as SUV_mean_ in the tumor. Tumor-to-heart ratios were 1.4 ± 0.23 at baseline, which decreased to 0.95 ± 0.18 at 14 days (*p* > 0.05) but decreased significantly relative to baseline to 0.51 ± 0.07 at 28 days (*p* = 0.0163) post-treatment initiation (Figure 5C). As expected, the MDA-MB-468 xenografted mice responded to CDX-011 therapy (Figure 5D).

For the dasatinib plus CDX-011 combination-treated group, the tumor SUV_mean_ of [^89^Zr]Zr-DFO-CR011 in the tumor was 3.1 ± 0.46 at baseline, which increased to 4.3 ± 0.62 at 14 days (*p* = 0.0002) then decreased to 1.9 ± 0.14 at 28 days (*p* < 0.001) post treatment initiation (Figure 5B). Tumor-to-heart ratios were 1.7 ± 0.36 at baseline, which increased to 2.5 ± 0.74 at 14 days (*p* = 0.0266), then decreased to 0.85 ± 0.14 at 28 days (*p* = 0.0002) post treatment initiation (Figure 5C). Statistical analyses were based on a two-way ANOVA. Similarly to the dasatinib-treated group, the combination-treated group demonstrated the upregulation of gpNMB at 14 days post treatment (Figure 5B,C). These results collectively demonstrate that PET imaging with [^89^Zr]Zr-DFO-CR011 can quantify the 52.5% and 42.2% increase in gpNMB upregulation at 14 days post-treatment initiation relative to baseline for dasatinib and dasatinib plus CDX-011 combination treated MDA-MB-468 xenograft models, respectively.

### 3.4. Combination Treatment with CDX-011 and Dasatinib Was More Effective in Tumor Shrinkage Than Monotherapy in MDA-MB-468 Xenograft Models at Endpoint

Throughout the treatment period of dasatinib (n = 8), CDX-011 (n = 8), dasatinib plus CDX-011 (n = 8), or vehicle control (n = 6), tumor volumes of mice with MDA-MB-468 xenografted mice were measured twice per week. While no PET imaging was performed on the vehicle control group, these tumor volumes were compared with those of the treated groups to assess the efficacy of treatment. The percentage change in tumor volume (%CTV) relative to baseline over the treatment period was calculated and used as a measure of the effectiveness of treatment, where an increase in % CTV indicates tumor growth and a decrease in % CTV indicates tumor shrinkage (Figure 5D). Throughout the treatment period, the tumors from control mice continued to grow. At endpoint, there was a 102 ± 30% increase in the % CTV relative to baseline. In contrast, xenografted mice treated with dasatinib, CDX-011, or dasatinib plus CDX-011 combination showed varying patterns of tumor inhibition, ultimately all decreasing in size. At the endpoint, the % CTV were −22.9 ± 11.6% for dasatinib alone, −25.1 ± 10.5% for CDX-011 alone, and −54.0 ± 13.6% for dasatinib plus CDX-011 combination treatment groups. All treatment groups showed a significant decrease in tumor volume compared to the vehicle control group (*p* < 0.0001). There was no difference in % CTV between the groups treated with single-agent dasatinib or CDX-011. The dasatinib plus CDX-011 combination treatment was more effective than either dasatinib (*p* < 0.05) or CDX-011 (*p* < 0.05) monotherapies at the endpoint (Kruskal–Wallis test). Finally, the health of the mice for the duration of imaging and treatment studies was constant with a BCS score of 3 (well-maintained).

### 3.5. Dasatinib Did Not Affect the Tumor Uptake of [^89^Zr]ZrDFO-CR011 in gpNMB-Negative MDA-MB-231 Xenograft Models

**Figure 6:**
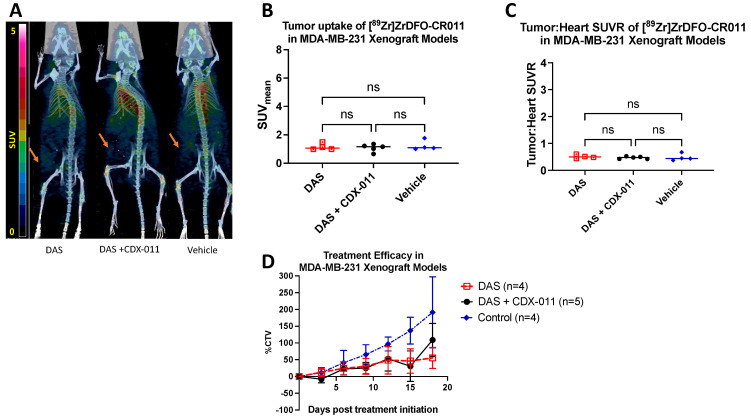
Dasatinib does not significantly affect [^89^Zr]Zr-DFO-CR011 uptake in MDA-MB-231 xenografted tumors at 21 days post treatment initiation (ns, *p* > 0.05). (**A**) Representative PET/CT images rendered as maximum intensity projections from each treatment group. Arrows point to tumor. RapID ear tags are shown as white squares. (**B**) Quantification of [^89^Zr]Zr-DFO-CR011 uptake in the tumor as SUV_mean_ and (**C**) Tumor-to-Heart SUVR after treatment (n = 4–5 per group). (**D**) Change in tumor size measured as the percentage of change in tumor volume (% CTV) relative to baseline after treatment initiation (n = 4–5 per group). DAS = dasatinib. Dasatinib treatment reduced tumor volume but not in a statistically significant way compared with those treated with vehicle control or combination of CDX-011 and dasatinib during the 21-day treatment period (*p* > 0.05).

MDA-MB-231 (gpNMB-negative) xenografted mice were used as a negative control for PET imaging with [^89^Zr]Zr-DFO-CR011. MDA-MB-231 xenografts were treated with (1) dasatinib alone (n = 4), (2) dasatinib plus CDX-011 combination (n = 5), or (3) vehicle control (n = 4). A single PET imaging with [^89^Zr]Zr-DFO-CR011 was performed 21 days after treatment (endpoint). This endpoint was shorter than that of MDA-MB-468 models due to the tumor burden in the vehicle control group. Figure 2 illustrates the PET imaging and treatment paradigm in this tumor model. Representative PET images from each group are shown as maximum intensity projections (Figure 6A). Figure S5 shows the uptake of the tracer in normal organs over time. Dasatinib treatment did not affect tracer uptake in the tumor, and there was no difference between the SUV_mean_ in the tumor or tumor-to-heart ratios for the different treatment groups (*p* > 0.05, Figure 6B,C). SUV_mean_ in the tumor was 1.2 ± 0.24 for dasatinib, 1.1 ± 0.27 for the combination therapy, and 1.2 ± 0.36 for the vehicle control group. The tumor-to-heart ratio was 0.51 ± 0.07 for dasatinib monotherapy, 0.47 ± 0.05 for the combination therapy, and 0.48 ± 0.13 for the vehicle control group (*p* > 0.05). The treatment also did not significantly affect tumor volumes which were measured twice per week throughout the 21-day treatment period. At the endpoint, there was a 55.5 ± 31.3% increase for dasatinib-treated group, a 109 ± 49% increase for the combination therapy, and a 190 ± 106% increase in % CTV for the vehicle control group (*p* > 0.05, Figure 6D). There was no significant difference in % CTV relative to baseline between the treatment groups (*p* > 0.05).

## 4. Discussion

### 4.1. Monitoring gpNMB Expression via Immunopet with [^89^Zr]Zr-DFO-CR011

ImmunoPET imaging has the potential to overcome the limitations of tissue-based biopsy analyses to determine antigen expression over the course of targeted treatment. Tissue-based biopsies would require multiple consecutive biopsies to obtain tissue specimens, some of which cannot be obtained due to their depth and location. In contrast, immunoPET imaging could be performed longitudinally and provide information on antigen expression over the course of treatment. As a proof-of-concept, we performed longitudinal immunoPET imaging with [^89^Zr]Zr-DFO-CR011 to measure the expression of gpNMB on the surface of TNBC cells, which is upregulated by tyrosine kinase inhibitors such as dasatinib. The mechanism by which dasatinib upregulates gpNMB expression has yet to be fully elucidated but is outside the scope of this study. Our goal is to identify the timepoint at which to administer the anti-gpNMB CDX-011. This approach is a rational design that enhances drug delivery by exploiting the upregulation of gpNMB in noninvasive and quantitative interventions. Furthermore, combining the CDX-011 with dasatinib has the potential to overcome resistance to the CDX-011 monotherapy through lower dosing and the combination of different mechanisms of action that inhibit cancer signaling pathways to effectively kill tumor cells.

We hypothesized that the tumor uptake of [^89^Zr]Zr-DFO-CR011 will increase in gpNMB-positive MDA-MB-468 xenograft models after treatment with dasatinib relative to baseline imaging. Longitudinal immunoPET imaging with [^89^Zr]Zr-DFO-CR011 in gpNMB-positive MDA-MB-468 xenograft models indicated that gpNMB was upregulated at 14 days post treatment initiation with dasatinib alone or with dasatinib and CDX-011 combination through the increase in tumor uptake of the tracer relative to baseline scans (Figure 5A–C). This result concurs with Western blot data that confirm increased gpNMB expression at 14 days post treatment initiation with dasatinib (Figure 4B). Longitudinal immunoPET imaging of gpNMB expression is feasible, given that [^89^Zr]Zr-DFO-CR011 internalizes in the tumor cell rapidly after binding to gpNMB on the cell surface [24]. Potential blocking of gpNMB from the first injection of the tracer was not apparent by immunoPET imaging in the second injection of the tracer, given that SUV measurements between the two imaging sessions were not significantly different (Figure S3).

We also evaluated the effects of longitudinal immunoPET imaging on tracer uptake in normal organs. Tracer uptake in various organs such as the liver, lung, and heart was quantified as SUV_mean_ in the same cohort of MDA-MB-468 xenografted mice at baseline, 14, and 28 days post treatment initiation with dasatinib alone, CDX-011 alone, or combination of the two. Tracer uptake increased significantly at 28 days post treatment initiation in the heart, lung, and liver but not bone. This effect could be due to increased bioavailability of the tracer in the presence of the ADC or multiple injections of the tracer. Another possibility could be tracer binding to shed gpNMB from tumor cells, but a limitation of our study is that we did not determine gpNMB concentration in the blood at the endpoint, and it should be explored further in future studies. Increased tracer uptake in the heart is not sufficient to delineate whether the tracer bound to shed gpNMB in the blood or the bioavailability of the tracer increased in the presence of CDX-011 (Figure S4). Discordance between SUV_mean_ in the tumor and tumor-to-heart SUVR, where SUV_mean_ remained constant from 14 to 28 days post treatment initiation and tumor-to-heart SUVR decreased, indicates the possibility of shed gpNMB or increased bioavailability (Figure 5B,C). Nevertheless, the imaging metrics used in longitudinal immunoPET studies need to be carefully evaluated. A decrease in tumor-to-heart SUVR over time may not mean a decrease in antigen expression. Finally, there was no significant effect in tracer uptake in these organs as a function of treatment type (Figure S4), which suggests that dasatinib does not alter the biodistribution of CDX-011 in normal organs during combination therapy.

### 4.2. Rational Design for Combination Therapy

We exploited the upregulation of gpNMB to explore the efficacy of combination treatment with dasatinib and CDX-011. We hypothesized that gpNMB upregulation through dasatinib treatment would enhance therapeutic efficacy with CDX-011. We compared the effects of dasatinib and CDX-011 in combination with both single-agent therapies on tumor growth. Combination treatment exerted a synergistic therapeutic effect in MDA-MB-468 xenografts over single-agent treatment at the study endpoint (Figure 5D). This effect is supported by in vitro studies of combination treatment in MDA-MB-468 cells, which were determined to have a synergistic therapeutic effect by Combenefit software analysis (Figure S1). In this exploratory study, we did not test different doses in vivo, which is a limitation of this study.

Dasatinib induced gpNMB expression in MDA-MB-231 cells in vitro but was not enough to achieve a synergistic therapeutic effect in combination with CDX-011 in vitro and in vivo (Figure 3B and Figure 6D). Additionally, the MDA-MB-231 xenograft models had high tumor burden at 21 days post treatment initiation, which limited our investigation in comparing treatment effects in this tumor model with that of MDA-MB-468 models that had an endpoint of 28 days post treatment initiation.

More studies are needed to fully characterize and elucidate the mechanism of gpNMB upregulation by dasatinib treatment in TNBC. Pharmacologically, our study agrees with the study by Biondini et al., in which they demonstrate that small molecule inhibitors heat shock protein 90 (HSP90) inhibitors upregulate gpNMB expression via diminishing PI3K/AKT/mTOR signaling pathway [15]. Dasatinib has also been shown to suppress PI3K/AKT/mTOR, which could also be the mechanism for upregulating the expression of gpNMB on the cell surface [28,29]. This evidence is likely the mechanism by which dasatinib increases gpNMB expression and sensitizes tumor cells to CDX-011 therapy and warrants further investigation to test this hypothesis. In the current study, we demonstrated that changes in gpNMB expression can be monitored in vivo via immunoPET and that the timepoint for gpNMB upregulation is 14 days post treatment initiation with dasatinib. ImmunoPET with [^89^Zr]Zr-DFO-CR011 could be used as an imaging tool to evaluate gpNMB upregulation with other strategies for combination treatment like that of HSP90 inhibitors and improve ADC delivery to tumor cells at the right time.

## 5. Conclusions

Dasatinib can upregulate gpNMB expression in the MDA-MB-468 cell line with moderate gpNMB expression cell line but can induce expression in gpNMB-negative MDA-MB-231 cell lines in vitro. The dasatinib and CDX-011 combination treatment significantly reduced the change in tumor volumes in MDA-MB-468 xenografted mice, while it did not have significant effects in the gpNMB-negative MDA-MB-231 models. PET imaging of gpNMB expression with [^89^Zr]Zr-DFO-CR011 has the potential to identify the timeframe for combination treatments via sequential administration of therapies that upregulate gpNMB expression and deliver cytotoxic payload through gpNMB targeting.

## Data Availability

The data presented in this study are available in this article (and supplementary material) and upon request from corresponding authors.

## Supplementary Materials

The following are available online at https://www.mdpi.com/article/10.3390/cancers15051589/s1, Figure S1: Dasatinib and CDX-011 have a synergistic therapeutic effect in gpNMB-positive MDA-MB-468 cells in vitro but not in gpNMB-negative MDA-MB-231. Figure S2: Full Western blot images showing the ratio of band intensities between gpNMB, Src, or p-Src relative to β-actin. Figure S3: Serial injection of MDA-MB-468 xenografted mice with [^89^Zr]ZrDFO-CR011 does not affect the results of tracer uptake in the tumor. Figure S4: Uptake (SUV_mean_) of [^89^Zr]ZrDFO-CR011 in normal organs of MDA-MB-468 xenografted mice over time following treatment with dasatinib (DAS), CDX-011, or the combination of the two. Figure S5: Uptake (SUV_mean_) of [^89^Zr]ZrDFO-CR011 in normal organs of MDA-MB-231 xenografted mice following treatment with DAS, its combination with CDX-011, or vehicle control.
