# Supplementary material for: ImmunoPET Imaging Identifies the Optimal Timepoint for Combination Therapy in Xenograft Models of Triple-Negative Breast Cancer
Source: Cancers (Basel). 2023 Mar 3;15(5):1589. doi: 10.3390/cancers15051589 (PMC10001369; doi:10.3390/cancers15051589)
Supplement: Supplementary file 1 [file cancers-15-01589-s001.zip › cancers-2179500-supplementary.pdf]

Supplementary Methods

Cell viability

One hundred microliters of MDA-MB-468 or MDA-MB-231 cells were seeded in a 96-well plate at 5,000 cells/well and incubated in a humidified incubator (at 37°C, 5% CO_2) for 24 h. A stock concentration of 10 mM of dasatinib (BMS-354825, CAS No. 302962-49-8, SelleckChem.com cat# S1021) in 50% polypropylene glycol in water was diluted in complete medium prior to adding to cells. First, we evaluated the sensitivity of dasatinib in these cell lines by determining the IC50 values of dasatinib for each cell line. Cells were then exposed to 10 μL of various concentrations dasatinib and incubated at 37°C with 5% CO_2 for 48 h. Viability of the cells were measured by adding 10 μL of CCK-8 reagent (Abcam cat# ab228554) and incubating for 3 h followed by measuring the optical density at 450 nm (OD_{450nm}) using a microplate reader (Biotek). IC50 values for dasatinib were determined by plotting the OD_{450nm} values against the concentration of dasatinib added, followed by fitting with a nonlinear regression curve ([Inhibitor] vs. normalized response -- Variable slope) using Graphpad Prism software v.8.

Dose finding study. For combination treatment with dasatinib and the anti-gpNMB antibody drug conjugate, CDX-011, both cell lines were plated in a tissue culture treated 96-well plate as described above. Different concentrations of dasatinib (0 - 8.3 μM) going down the columns and CDX-011 (0 - 2.8 μM) going across the rows of the plate were added and incubated at 37°C with 5% CO_2 for 48 h. Viability was measured as described above. Drug combination effects were analyzed using Combenefit software (http://sourceforge.net/projects/combenefit/). PBS was used as a control, from which cell viability was normalized as % Control.

Supplementary Results

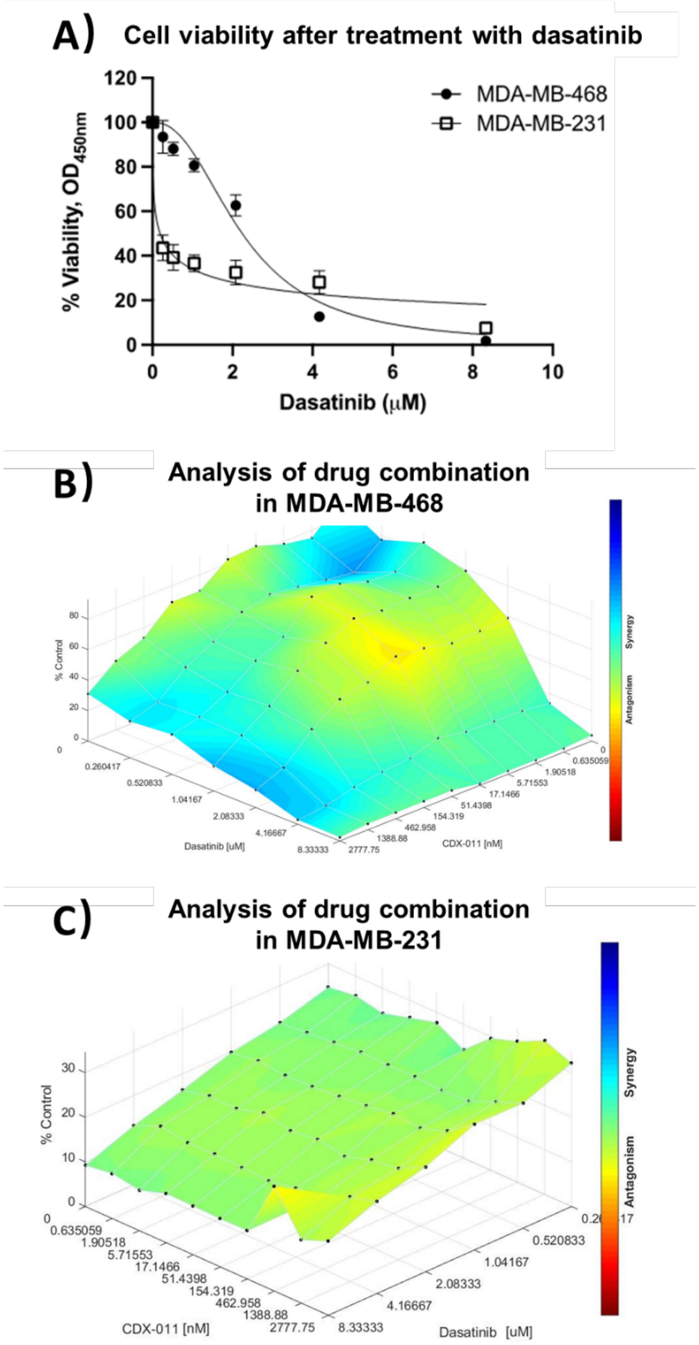

Figure S1. Dasatinib and CDX-011 have a synergistic therapeutic effect in gpNMB-positive MDA-MB-468 cells in vitro but not in gpNMB-negative MDA-MB-231. A) Dasatinib has higher potency in MDA-MB-231 cells compared with MDA-MB-468 cells with IC_{50} of 0.19 μM (95% CI of 0.069 – 0.33) for MDA-MB-231 and 2.3 μM (95% CI of 2.0 – 2.5) for MDA-MB-468. B) Three-dimensional plot of dasatinib and CDX-011 against the percentage of cell viability in MDA-MB-468 cells and in C) MDA-MB-231 cells.

Concentrations of 4 μM of dasatinib with 1.3 μM of CDX-011 provided at least 50% decrease in cell viability (Fig. S1B). As expected, there was no synergistic therapeutic effect in gpNMB-negative MDA-MB-231 cells (Fig. S1C). Only dasatinib contributed to killing MDA-MB-231 cells *in vitro*. Plots in B and C were analyzed using Combenefit program (http://sourceforge.net/projects/combenefit/). Blue regions represent the concentrations of dasatinib and CDX-011 with synergistic effect. These concentrations were used for in vivo studies.

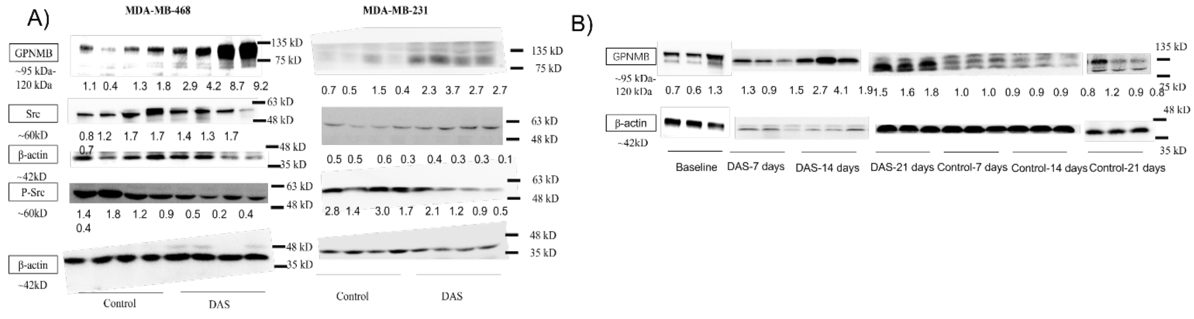

Figure S2: Full western blot images showing the ratio of band intensities between gpNMB, Src, or p-Src relative to β-actin. A) Treatment with dasatinib (DAS) or vehicle control in MDA-MB-468 and MDA-MB-231 cells in vitro after 48 h. B) Tumor lysates after treatment of MDA-MB-468 xenografted mice with dasatinib or vehicle control over time.

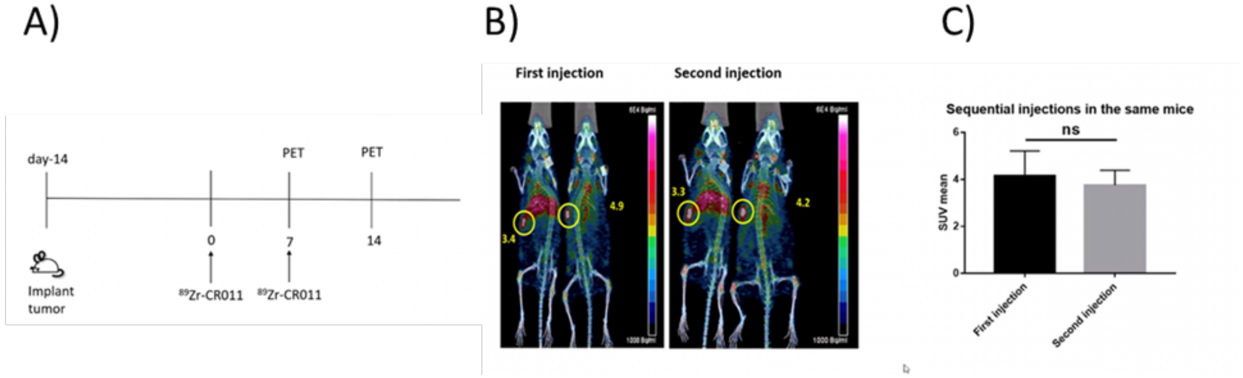

Figure S3. Serial injection of MDA-MB-468 xenografted mice with [^{89}Zr]Zr-DFO-CR011 does not affect the results of tracer uptake in the tumor. A) Timeline of tracer injections and sequential PET imaging. B) Maximum intensity projections of representative mice; C) there was no statistically significant difference in the SUV_{mean} of these two PET scans ($p > 0.05$, based on t-test).

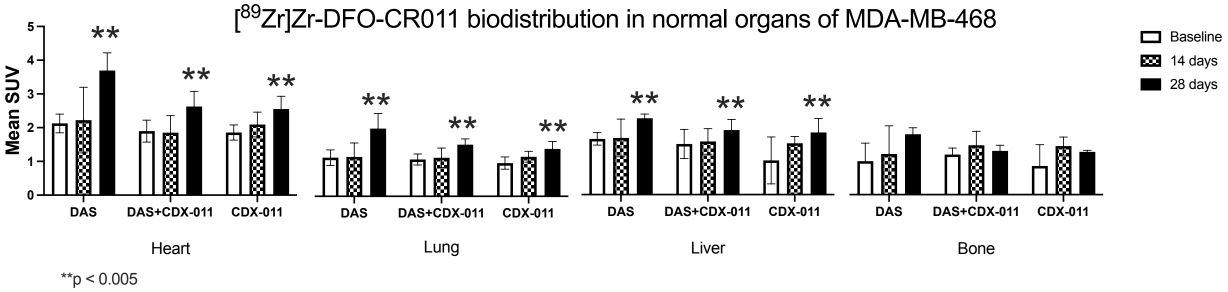

Figure S4: Uptake (SUV_{mean}) of [^{89}Zr]Zr-DFO-CR011 in normal organs of MDA-MB-468 xenografted mice over time following treatment with dasatinib (DAS), CDX-011, or combination therapy of the two. Tracer uptake is significant at the 28-day time point compared with that in earlier timepoints post treatment initiation in the heart, lung, and liver (**$p < 0.005$ based on 2-way ANOVA). Treatment effects on tracer uptake in each organ are not significant ($p > 0.05$).

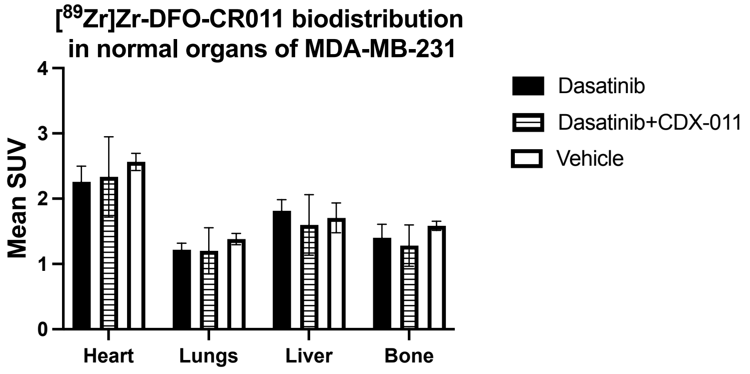

Figure S5: Uptake (SUV_{mean}) of [^{89}Zr]Zr-DFO-CR011 in normal organs of MDA-MB-231 xenografted mice following treatment with DAS, its combination with CDX-011, or vehicle control. There are no statistically significant differences between the treatment groups for each organs ($p > 0.05$ based on one-way ANOVA).
